# Characterization of grapevine leafroll-associated virus 3 genetic variants and application towards RT-qPCR assay design

**DOI:** 10.1371/journal.pone.0208862

**Published:** 2018-12-12

**Authors:** Alfredo Diaz-Lara, Vicki Klaassen, Kristian Stevens, Mysore R. Sudarshana, Adib Rowhani, Hans J. Maree, Kar Mun Chooi, Arnaud G. Blouin, Nuredin Habili, Yashu Song, Kamyar Aram, Kari Arnold, Monica L. Cooper, Lynn Wunderlich, Mark C. Battany, Larry J. Bettiga, Rhonda J. Smith, Rachelle Bester, Huogen Xiao, Baozhong Meng, John E. Preece, Deborah Golino, Maher Al Rwahnih

**Affiliations:** 1 Department of Plant Pathology, University of California-Davis, Davis, California, United States of America; 2 Foundation Plant Services, University of California-Davis, Davis, California, United States of America; 3 Department of Evolution and Ecology, University of California-Davis, Davis, California, United States of America; 4 United States Department of Agriculture, Agriculture Research Service, University of California-Davis, Davis, California, United States of America; 5 Department of Genetics, Stellenbosch University, Stellenbosch, South Africa; 6 The New Zealand Institute for Plant and Food Research Limited, Auckland, New Zealand; 7 School of Agriculture, Food and Wine, The University of Adelaide, Adelaide, Australia; 8 Department of Molecular and Cellular Biology, University of Guelph, Guelph, Ontario, Canada; 9 University of California, Cooperative Extension-Stanislaus County, Modesto, California, United States of America; 10 University of California, Cooperative Extension-Napa County, Napa, California, United States of America; 11 University of California, Cooperative Extension-Central Sierra, Placerville, California, United States of America; 12 University of California, Cooperative Extension-San Luis Obispo County, San Luis Obispo, California, United States of America; 13 University of California, Cooperative Extension-Monterey County, Monterey, California, United States of America; 14 University of California, Cooperative Extension-Sonoma County, Sonoma, California, United States of America; 15 National Clonal Germplasm Repository, United States Department of Agriculture, Agricultural Research Service, Davis, California, United States of America; Universita del Salento, ITALY

## Abstract

Grapevine leafroll-associated virus 3 (GLRaV-3) is the most widely prevalent and economically important of the complex of RNA viruses associated with grapevine leafroll disease (GLD). Phylogenetic studies have grouped GLRaV-3 isolates into nine different monophyletic groups and four supergroups, making GLRaV-3 a genetically highly diverse virus species. In addition, new divergent variants have been discovered recently around the world. Accurate identification of the virus is an essential component in the management and control of GLRaV-3; however, the diversity of GLRaV-3, coupled with the limited sequence information, have complicated the development of a reliable detection assay. In this study, GLRaV-3 sequence data available in GenBank and those generated at Foundation Plant Services, University of California-Davis, was used to develop a new RT-qPCR assay with the capacity to detect all known GLRaV-3 variants. The new assay, referred to as FPST, was challenged against samples that included plants infected with different GLRaV-3 variants and originating from 46 countries. The FPST assay detected all known GLRaV-3 variants, including the highly divergent variants, by amplifying a small highly conserved region in the 3’ untranslated terminal region (UTR) of the virus genome. The reliability of the new RT-qPCR assay was confirmed by an enzyme linked immunosorbent assay (ELISA) that can detect all known GLRaV-3 variants characterized to date. Additionally, three new GLRaV-3 divergent variants, represented by four isolates, were identified using a hierarchical testing process involving the FPST assay, GLRaV-3 variant-specific assays and high-throughput sequencing analysis. These variants were distantly related to groups I, II, III, V, VI, VII and IX, but much similar to GLRaV-3 variants with no assigned group; thus, they may represent new clades. Finally, based on the phylogenetic analysis, a new GLRaV-3 subclade is proposed and named as group X.

## Introduction

Grapevine leafroll-associated virus 3 (GLRaV-3) is the most important virus pathogen of grapevine worldwide and the main etiological agent of grapevine leafroll disease (GLD) [1]. GLRaV-3 can be transmitted by grafting and by mealybugs (Homoptera: Pseudococcidae) and/or soft scale insects (Homoptera: Coccidae) [2,3]. However, the long-distance spread of GLRaV-3 is largely mediated by the movement of infected budwood by growers. The economic benefits from the provision of GLRaV-3 certified virus-free planting stock is valued at US $53.5 million annually for the North Coast of California alone [4].

A recent study by Maree et al. [5] based on genome wide phylogenetic analysis of complete and incomplete genomes demonstrated that species *Grapevine leafroll-associated virus 3* (genus *Ampelovirus*; family *Closteroviridae*) can be divided into eight distinct subclades (groups I-VIII) and four supergroups (supergroups A-D). Initially, isolates of GLRaV-3 were classified in five groups (I–V) [6,7]; later, the discovery of highly divergent variants, distantly related to groups I–V, led to the creation of groups VI and VII [8–10]. Supergroup A includes groups I–V while supergroups B–D include groups VI, VII, and VIII, respectively [5]. Group VIII is consisted of isolates from Portugal, however, their sequence data was recently removed from GenBank. Lastly, a recent published paper described a novel genetic variant of GLRaV-3 from the USA and proposed the creation of a new phylogroup (group IX) [11]. Classification of GLRaV-3 isolates has been done largely by phylogenetic analysis of the genes encoding coat protein (CP), 70 kDa heat shock protein homologue (HSP70h), or RNA-dependent RNA polymerase (RdRp) because of the limited availability of whole genome sequence information for many isolates [1].

The distribution of GLRaV-3 variant groups has been studied in different regions of the world. In South Africa, groups I, II, III, VI, and VII were identified, but groups II and VI were found to be more predominant [12]. In New Zealand, groups I and VII were predominant among isolates found in commercial vineyards and germplasm repository [13]. In China and Portugal, there was a high incidence of GLRaV-3 variants belonging to group I [14,15]. Genetically diverse members of groups I-VI were identified in Napa Valley, CA, by amplifying regions of the CP gene and the 3’ terminal region [9,10]. Sharma et al. [9] concluded that in the Napa Valley, variant group I was predominant followed by groups II and III; furthermore, mixed variant infections by group I and III were common. In addition, two new variants were identified in the Napa Valley survey [9]. The first one, GLRaV-3e, was similar to a divergent isolate from New Zealand (NZ-1) and the second variant, GLRaV-3f, shared only about 75% identity to isolates in groups VII and VIII and 80% identity to isolates in supergroup A, indicating that it might represent a new GLRaV-3 group.

More recent studies in California [16] reconstructed nearly complete genome sequences of additional GLRaV-3 variants, some of which had not been previously reported in the USA. Grapevines that tested negative with a reverse transcription quantitative PCR (RT-qPCR) assay targeting all group I-V and GLRaV-3e variants were analysed by high-throughput sequencing (HTS) that led to the discovery of three divergent GLRaV-3 variants. Isolate TRC139 (GenBank: KY764332) has 99% nucleotide (nt) identity to NZ2, a highly divergent GLRaV-3 group VI variant reported only in New Zealand [8]. Isolate NdA121 (GenBank: KY707826) was closely related to GH24 (88% identity), the divergent variant which was first identified in South Africa [5]. Isolate TRC138 (GenBank: KY764333) was similar to the partial sequence of GLRaV-3f (100% identity), the variant identified first in the Napa Valley [9].

Starting in 2016, as part of the new introduction testing protocol at Foundation Plant Services (FPS), University of California-Davis, grapevine plants have been analyzed by HTS to determine the phytosanitary status before inclusion in the FPS collection. As a result, 34 near-complete genome sequences of GLRaV-3 were obtained and deposited in publicly available databases. The nt sequences of these isolates exhibited considerable sequence identities with known variants (groups I, II, III and VII) or with highly divergent variants [16]. The GLRaV-3 isolates were obtained from different grapevine cultivars originating in Greece, Hungary, Italy, Spain, Canada and the USA, confirming their wide-spread distribution.

Although enzyme linked immunosorbent assay (ELISA) and end-point RT-PCR assays have been the preferred high-throughput testing methods for GLRaV-3 and other economically important grapevine viruses, RT-qPCR has become a standard tool for virus detection given its speed, specificity and sensitivity [17]. However, designing a reliable GLRaV-3 RT-qPCR test has been complicated by the fact that GLRaV-3 is genetically highly diverse. The original GLRaV-3 RT-qPCR assay used at the FPS was designed to amplify all group I-V variants [18]. Later, an assay specific to GLRaV-3e was added, to detect group VI variants. However, even with this multiplex assay, highly divergent variants with degeneracies in the primer regions were not detected during the testing process.

With the identification of additional GLRaV-3 variants and the availability of complete or nearly complete genome sequences for many of these variants, it has become feasible to identify conserved regions and design a RT-qPCR assay that can detect all characterized GLRaV-3 variants. In this paper, we report a new RT-qPCR assay, referred to as FPST, which amplifies a conserved 3’ terminal region of the virus genome. Our assay was challenged against a high number of GLRaV-3-infected samples; and the detection was confirmed by double antibody sandwich (DAS)-ELISA assay using an in house-developed antiserum. Subsequently, a combination of different RT-qPCR assays (including the FPST) and HTS analysis was employed to identify potentially new variants and the GLRaV-3 genetic diversity is discussed.

## Materials and methods

### RT-qPCR assay detecting GLRaV-3 genetically diverse variants

Forty-three GLRaV-3 sequences, including 20 complete genome and 23 near-complete genome sequences deposited in the GenBank database, were used for assay design. These sequences included newly characterized GLRaV-3 variants from FPS and two distinct variants recently reported in Canada [19]. All the sequences were aligned using MUSCLE [20], and Geneious v10.2.2 (Biomatters, New Zealand) was used to identify regions with low sequence diversity and therefore, suitable for assay design.

Primer Express software (Applied Biosystems, Foster City, CA, USA) was employed to design six primers and two MGB probes with FAM as the 5’ reporter. Specificity was verified using the PrimerBLAST software (http://www.ncbi.nlm.nih.gov/tools/primer-blast/). RT-qPCR reactions were done in the QuantStudio 6 real-time PCR system using the TaqMan Fast Virus 1-Step Master Mix from ThermoFisher Scientific (Foster City, CA, USA) according to the manufacturer’s specifications. Each reaction (10 μl) included 2 μl of total nucleic acid (TNA) and final primer and probe concentrations of 900 and 250 nM, respectively. Reverse transcription and amplification conditions were as follows: 50 °C for 5 min, 95 °C for 20 s, followed by 40 cycles of 95 °C for 3 s and 60 °C for 30 s. Assay efficiency was determined using serial dilutions (1:1 to 1:1,000,000) of GLRaV-3 isolate NY1 TNA in water; the initial concentration was 10 ng/μl of TNA.

The new RT-qPCR assay (FPST assay) was first empirically tested using grapevines already known to have single-variant infections by isolates similar to the following GLRaV-3 isolates: GH24, NZ2, 43–15 (variant GLRaV-3f), 7–1006 (variant GLRaV-3e) and NY1, the last-mentioned belonging to a common variant group in California. These plants are used as routine positive controls in the FPS testing process and they are maintained under a strict vector control program. Such plants were previously analyzed by HTS to determine the phytosanitary status. On the other hand, a healthy grapevine and grapevines free of GLRaV-3, but infected with grapevine leafroll-associated virus 1 (GLRaV-1), grapevine leafroll-associated virus 2 (GLRaV-2), grapevine leafroll-associated virus 4 (GLRaV-4), GLRaV-4 strain 5, GLRaV-4 strain 6 and GLRaV-4 strain 9 based on HTS, were tested with the FPST assay to investigate any cross-reaction event.

### Plant material and large-scale testing by the FPST assay

During the fall of 2017, a total of 2,415 plant samples (S1 Table) were collected from grapevine populations with a historically high incidence of GLRaV-3 or with observable GLD symptoms such as interveinal reddening and downward rolling of leaf margins in red wine cultivars, and interveinal chlorosis and downward rolling of leaf margins in white cultivars [1]. These grapevine populations included: 1) the USDA National Clonal Germplasm Repository (NCGR) in Winters, CA, in which a previous study [18] identified plants infected with GLRaV-3 and originating from 12 different countries (1,206 samples); 2) the Davis Virus Collection (DVC) at University of California-Davis [21], which primarily consists of domestic GLRaV-3 isolates and is also the source of an isolate which is 99% identical to the GH24 isolate (109 samples); 3) the FPS pipeline of foreign and domestic introductions (417 samples); and 4) 89 vineyards in the main grape-growing areas of California including 77 samples from Napa Valley, 14 samples from Sonoma, 44 samples from San Luis Obispo, 39 samples from Monterey, 156 samples from Central Coast, 10 samples from Coachella Valley, 70 samples from the North Coast, 164 samples from the San Joaquin Valley and 109 samples from the Central Sierra region. Additionally, 45 samples from different GLRaV-3 collections outside of the USA were included in this study. These included 23 South African grapevines from eight different selections that represent the different GLRaV-3 variant groups present in that country; six samples originated from New Zealand, where several new GLRaV-3 isolates have been reported recently [8]; seven GLRaV-3 positive plants originated from Australia that showed mild leaf roll symptoms [22]; an asymptomatic GLRaV-3 infected ‘Pinot noir’ vine originating from Spain; and lastly, eight grapevine samples determined positive for GLRaV-3 by end-point RT-PCR and HTS during a study validating this technology for virus detection in Canada, including a sample (ON936) from a ‘Vidal Blanc’ vine infected with a putative new GLRaV-3 variant based on preliminary sequence analysis. Positive controls (described above) were included during the testing process, as well as a grapevine that tested negative by HTS for viruses and virus-like pathogens.

All the above-mentioned samples (leaf petioles or bark scrapings) were subjected to TNA extraction: 0.2 g plant tissue was homogenized in 2 ml of guanidine isothiocyanate lysis buffer (4 M guanidine isothiocyanate; 0.2 M sodium acetate, pH 5.0; 2 mM EDTA; 2.5% (w/v) PVP-40) and TNA extracts were prepared using a MagMAX-96 viral RNA isolation kit (Ambion, Austin, TX, USA) as per the manufacturer’s protocol. Subsequently, the integrity of RNA was verified using an 18S rRNA assay [18].

### Validation of FPST assay through DAS-ELISA

To verify the ability of the FPST assay to detect different GLRaV-3 variants, all the samples involved in the large-scale screening were re-tested by an in house-developed DAS-ELISA assay. Our DAS-ELISA assay has detected all known GLRaV-3 variants characterized to date and consists of a mixture of two polyclonal and three monoclonal antibodies generated from recombinant proteins. A cocktail of polyclonal antibodies (1:5,000 dilution) was used as the primary antibody and a cocktail of monoclonal antibodies (1:10,000 dilution) as the secondary antibody, which was combined with a Sigma-Aldrich goat anti-mouse IgG-alkaline phosphatase conjugate (1:20,000 dilution). Samples were tested in duplicate, including negative and positive controls. OD_405nm_ was read after 2, 4 and 24 h incubation with 4-nitrophenyl phosphate disodium salt hexahydrate. Samples with an average absorbance value greater than three times the average absorbance value of negative controls were considered to be serologically positive. For a full protocol of DAS-ELISA see [17].

Sample preparation for DAS-ELISA was completed as described by Rowhani [23]. Thus, 0.5 g plant tissue was homogenized in 10 vol ELISA grinding buffer (0.01 M sodium carbonate; 0.035 M sodium bicarbonate; 0.05% (w/v) Tween 20; 2% (w/v) PVP 40; 2% (w/v) BSA) using a Homex grinder (Bioreba, South Bend, IN, USA).

### Identification of novel GLRaV-3 variants and genetic diversity

Samples that were positive by the FPST assay during the large-scale testing were then screened using our group I-V and GLRaV-3e multiplex RT-qPCR assay that detects only isolates within these groups. Samples tested negative from the multiplex assay were selected and screened using three variant-specific RT-qPCR assays (Table 1) that amplify isolates similar to 43–15 (variant GLRaV-3f), GH24 or NZ2. Following the methodology described by Sharma et al. [9], primers and probes (i.e. MGB or TAMRA) for these assays were designed based on multiple sequence alignments that identified conserved regions in one of the three variants but with low sequence similarity compared with the other GLRaV-3 variants. The specificity of the new variant-specific assays was confirmed using the PrimerBLAST software and challenged against plants singly infected with other GLRaV-3 variants (positive control plants). RT-qPCR reaction conditions for the above-mentioned assays were the same as described for the FPST assay. Samples found to be positive by FPST but negative by the multiplex and the variant-specific RT-qPCR assays were prioritized for HTS analysis.

**Table 1:** Primers and probes designed for the detection of grapevine leafroll-associated virus 3.

Assay	Primer Name	Sequence (5’-3’)	5’ Reporter	Probe Type
**Group I-V**^a^	LR3_HSP70-F	GGGTCAAGTGCTCTAGTTAAGGTCA		
	LR3_HSP7070-R1	AAAGTGTCCACCAGTCTCAGTCC		
	LR3_HSP70-R2	AAAGTGTCCACCAATCTCAGTCC		
	LR3_HSP70-P1	TTGCCGCAGATATCTA	FAM	MGB
	LR3_HSP70-P2	TTGCCGCACATATC	FAM	MGB
	LR3_HSP70-P3	TTGCCGCAAATAT	FAM	MGB
**LR3e**^a^	LR3e_HSP70-F1	TCCAGTTATCGCGGTGATGAC		
	LR3e_HSP70-F2	TCCAGTTATCGCCGTTATGACTG		
	LR3e_HSP70-R	CCACGTCTTTACGCACTTTCG		
	LR3e_HSP70-P1	CGGTTCGAGTGCTCT	FAM	MGB
	LR3e_HSP70-P2	TGGTTCGAGCGCTCT	FAM	MGB
**LR3f**	LR3f_HSP70-P	TTTACCGCAGATATCTAAT	NED	MGB
	LR3f_HSP70-F	GGGTCGAGCGCTCTAACCA		
	LR3f_HSP70-R	AAAGTGTCCACTAATCGTAGGCCA		
**NZ2**	LR3NZ2_HSP70-F	TATAGCTGTGATGACCGGCG		
	LR3NZ2_HSP70-R	AGGTGTCTACTAGTCGCAAAC		
	LR3NZ2_HSP70-P	TGTTCGGTGGCGTGTGGGGC	FAM	TAMRA
**GH24**	LR3GH24_HSP70-F	GTGGTGGCCGTCATGAC		
	LR3GH24_HSP70-R	GTTAGAGTGTTAGTTAGTGTGTCCA		
	LR3GH24_HSP70-P	CGGTGTTCAGTGGCCTGTGGGG	FAM	TAMRA
**FPST**	LR3_FPST-F1	ACCTCACGGTTTAATACTCTGATATTTG		
	LR3_FPST-F2	ACCTCACGGTTTAACACTCTGATGT		
	LR3_FPST-F3	ACCTCACGGTTTAATACTCAGATATTTG		
	LR3_FPST-F4	ACCTCTCGGTTTAACACTCTGATGTT		
	LR3_FPST-P1	AATAAACGCCAAAATCCAA	FAM	MGB
	LR3_FPST-P2	AATAAACGCCGAAATC	FAM	MGB
	LR3_FPST-R1	GAGGCCCGCCTAGGTCC		
	LR3_FPST-R2	GAGGCCCGCCTAGGTAC		

^a^Assays used as multiplex.

HTS library construction and sequencing was performed as described by Al Rwahnih et al. [24]. Briefly, aliquots of TNA from source samples were subjected to cDNA library construction and later sequenced using the Illumina NextSeq 500 platform. Illumina reads were demultiplexed and adapter trimmed prior to analysis using Illumina bcl2fastq v2.20.0.422. Virus sequences were obtained by subsequent *de novo* assembly into contiguous consensus sequences (contigs) using SPAdes v3.11 [25]. Contigs were identified as GLRaV-3 if their top BLASTn or BLASTx hits to the National Center for Biotechnology Information (NCBI) protein and nt databases of plant viruses were taxonomically assigned to GLRaV-3. Long contigs (>15,000 nts) showing a considerable sequence identity (E-value < 1e-5; > 80% protein or nt identity) with their closest known GLRaV-3 variant were identified and deposited in the GenBank as near-complete genomes.

Independently of the search for new GLRaV-3 variants, 42 random samples which tested positive by the FPST assay from the NCGR (15 samples), FPS pipeline (14 samples) and different regions in California (13 samples) were also analyzed by HTS to determine the GLRaV-3 diversity present in these grapevine populations. Contigs (> 4,000 nts) generated from the above-mentioned samples were compared against the known GLRaV-3 isolates for their identification using the BLASTn program of the NCBI.

### Phylogenetic analysis of GLRaV-3 variants

To determine the evolutionary relationships among GLRaV-3 variants, including variants identified during this work, a maximum likelihood phylogenetic tree was generated using complete or near-complete genomes (above 16,000 nts) available in GenBank and/or sequenced at FPS. Thus, 65 GLRaV-3 sequences (S2 Table) were aligned using MUSCLE [20] and later analyzed using MEGA7 [26]; the phylogenetic analysis was performed using the Tamura-Nei model and 1,000 bootstrap replications.

### Small-scale replica of survey in New Zealand

To replicate the results of the large-scale testing conducted at FPS, a small survey was conducted at The New Zealand Institute for Plant and Food Research Limited in Auckland, New Zealand. Briefly, 61 grapevine samples were collected from diverse regions of New Zealand and tested by both the FPST assay and the DAS-ELISA developed at FPS. These samples included symptomatic and asymptomatic plants from multiple cultivars (white and red), and samples from a field trial with vines carrying three different GLRaV-3 variants (group I and VI, and variant NZ2) [27].

## Results

### RT-qPCR assay detecting genetically diverse variants

**Fig 1:**
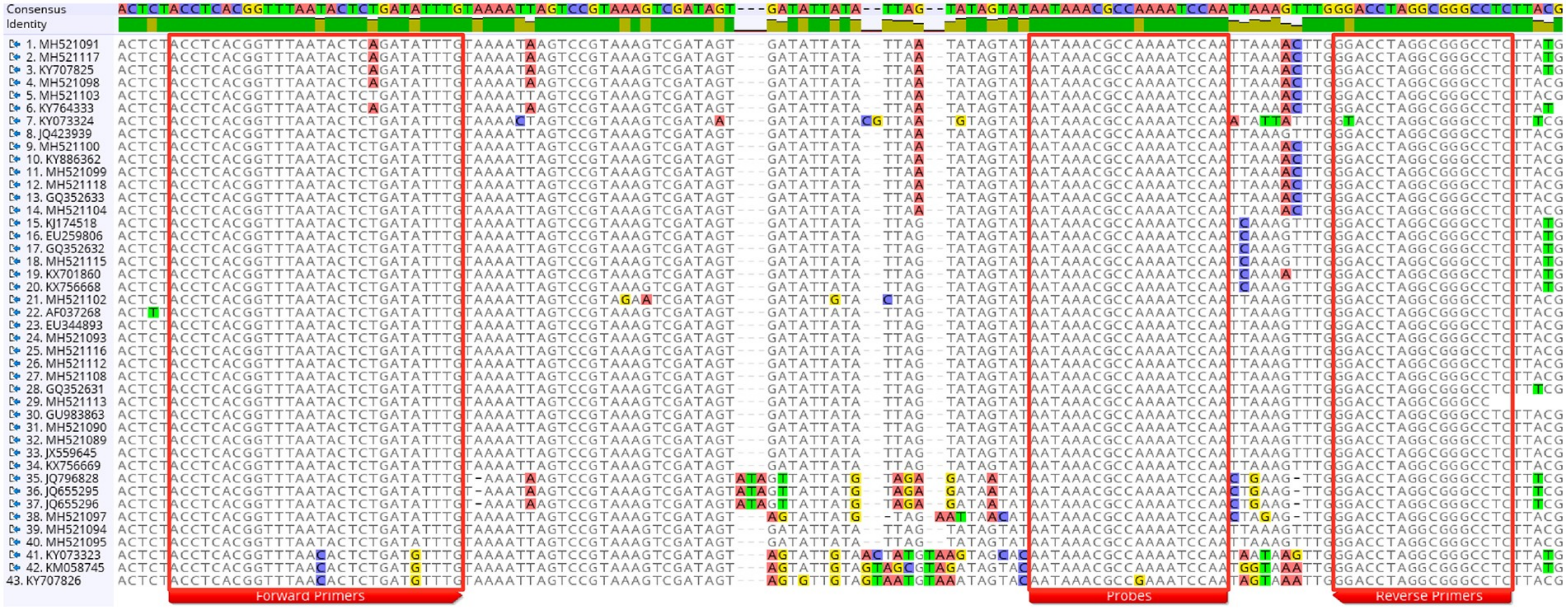
Alignment of the 3’ terminal region of grapevine leafroll-associated virus 3 (GLRaV-3). Selected regions for primer and probe design are identified by red squares. GLRaV-3 sequences were obtained from the GenBank or generated at FPS.

**Fig 2:**
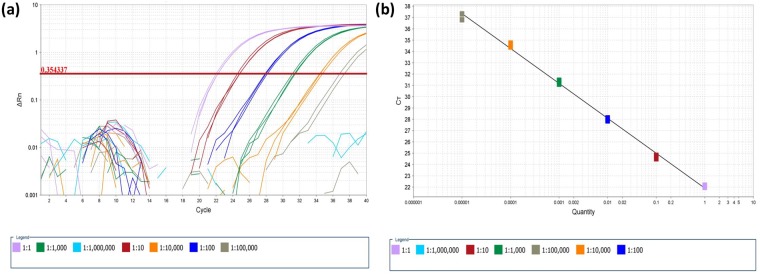
Relative grapevine leafroll-associated virus 3 (GLRaV-3) quantification of the FPST assay. (a) Amplification plot and (b) standard curve. Cycle threshold (C_T_) values obtained for three replicates of ten-fold serial dilutions of GLRaV-3 isolate NY1 control are plotted.

The genome-wide sequence alignment exhibited a small, but highly conserved region at the 3’ untranslated terminal region (UTR) of the GLRaV-3 genome (Fig 1). Consequently, the target for the new RT-qPCR assay is located inside the last 200 nts of the virus genome, hence the name of FPS Terminal or FPST assay was designated for the test. FPST is an assay formed by two different MGB probes, four forward primers and two reverse primers (Table 1), which amplify a 125–128 nt region depending on the variants. The standard curve indicated that the FPST assay can detect the presence of GLRaV-3 isolate NY1 up to a 1:100,000 dilution (Fig 2). The slope of the standard curve (-3.085) was used to calculate an amplification efficiency of 110.91% with a coefficient of correlation (R^2^) of 0.99.

**Fig 3:**
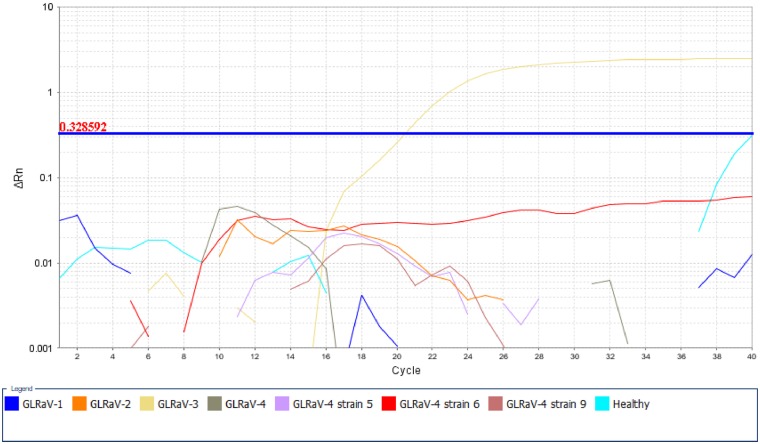
Amplification plot generated by the FPST assay on grapevines carrying different grapevine leafroll-associated virus (GLRaV) species and a healthy plant.

During the initial tests using positive control samples, the FPST assay detected all five different GLRaV-3 variant groups that we had available as single-variant infections, including the highly diverse variants GH24 and NZ2 (Table 2). Such detection was confirmed by triplicate samples, and in all cases the C_T_ values ranged from 19.5 to 24.6. Additionally, no cross-reaction was observed with other GLRaV species or a healthy plant (Fig 3).

**Table 2:** Detection of grapevine leafroll-associated virus 3 (GLRaV-3) isolates/variants by different RT-qPCR assays.

		RT-qPCR Assays (C_T_ values)				
		Group I-V + LR3e	LR3f	NZ2	GH24	FPST
**GLRaV-3** **Isolates**	**NY1**	+ (19.8) + (19.3) + (19.4)	- - -	- - -	- - -	+ (21.4) + (21.7) + (21.2)
	**7–1006** **(GLRaV-3e)**	+ (21.1) + (21.0) + (21.8)	- - -	- - -	- - -	+ (19.5) + (19.7) + (19.9)
	**43–15** **(GLRaV-3f)**	- - -	+ (25.6) + (25.6) + (25.7)	- - -	- - -	+ (24.0) + (24.4) + (24.6)
	**NZ2**	- - -	- - -	+ (18.5) + (18.3) + (18.7)	- - -	+ (22.6) + (22.3) + (22.9)
	**GH24**	- - -	- - -	- - -	+ (17.0) + (16.9) + (17.2)	+ (21.5) + (21.8) + (21.2)

Cycle threshold (C_T_); positive result (+); negative result (-).

### Large-scale testing by the FPST assay and DAS-ELISA validation

Fifty-six percent of the 2,460 samples, or 1,386 samples, tested positive for GLRaV-3 (S3 Table) by the FPST assay. These included 968 samples from NCGR, 68 samples from DVC, 128 samples from the FPS pipeline, and 177 samples from different grape growing regions of California; in addition, 45 samples received from collections out of the USA tested positive. At NCGR, positive vines originated from 43 different countries including the USA. Positive samples from the FPS pipeline included grapevines from the USA, Canada, Croatia, France, Greece, Hungary, Israel, Italy, South Korea, Portugal, Spain and Turkmenistan. Of the 177 positive samples from the grape growing regions of California, 41 were from Napa, three from Sonoma, 15 from San Luis Obispo, 35 from Monterey, 37 from the Central Coast, 10 from Coachella Valley, six from the North Coast, 21 from San Joaquin Valley and nine from the Central Sierra region. The FPST assay also confirmed the GLRaV-3-positive status of 45 samples belonging to collections from South Africa, New Zealand, Australia, Canada and Spain. Thus, the FPST assay detected GLRaV-3 in more than a thousand accessions that included both white and red fruit grapevines originating from 46 countries (Table 3). The FPST RT-qPCR produced C_T_ values that ranged from 17.4 to 30, with a mean of 21.2. Finally, all the samples tested positive by the FPST assay also tested positive by the in house-developed DAS-ELISA test, and similar agreement was obtained from the negative samples.

**Table 3:** Detection of grapevine leafroll-associated virus 3 by the FPST assay on grapevine samples from diverse origin.

Country of Origin	Positive Samples
Afghanistan	45
Algeria	1
Argentina	7
Australia	8
Austria	12
Belgium	4
Canada	47
Chile	3
China	1
Croatia	11
Denmark	2
Egypt	1
Former Serbia and Montenegro	6
France	35
Georgia	1
Germany	35
Greece	141
Hungary	7
India	6
Iran	4
Iraq	2
Israel	8
Italy	67
Japan	3
Kazakhstan	1
South Korea	4
Lebanon	1
Morocco	1
New Zealand	6
Pakistan	28
Peru	1
Portugal	34
Romania	2
Russian Federation	16
Serbia	8
South Africa	30
Soviet Union	20
Spain	10
Switzerland	1
Tunisia	1
Turkey	1
Turkmenistan	8
Ukraine	1
United Kingdom	1
United States of America	730
Yemen	1

### Characterization of novel GLRaV-3 variants and genetic diversity

**Fig 4:**
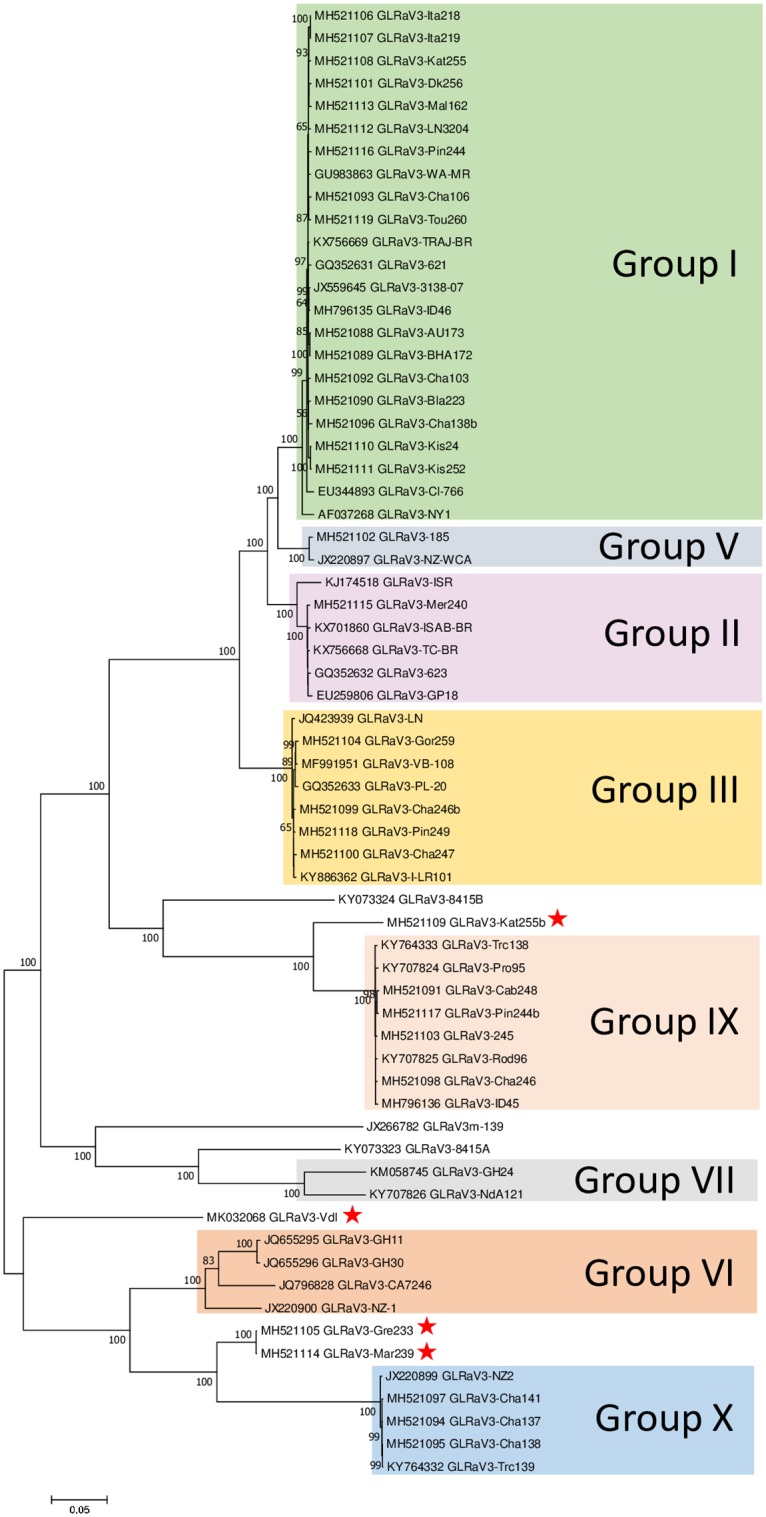
Phylogenetic inference of grapevine leafroll-associated virus 3 (GLRaV-3) isolates. Maximum likelihood tree generated based on complete or near-complete genomes (above 16,000 nts) available in GenBank and/or the FPS database. Bootstrap values less than 50% are not shown. Horizontal branch length is proportional to genetic distance and the scale bars represent changes per site. Red stars denote the divergent variants identified in this study.

Of the 1,386 FPST assay-positive samples, 1,327 (95%) samples also tested positive by the group I-V and GLRaV-3e multiplex RT-qPCR assay, indicating that these vines were infected with variants from these groups. When the 59 negative samples were tested with variant-specific assays, 13, 35 and seven samples were positive for variants GLRaV-3f, GH24 and NZ2, respectively. Consequently, four samples (including sample ON936) tested positive by the FPST assay but negative by the multiplex and variant-specific RT-qPCR assay; later, these samples were analyzed by HTS, generating sequence data with considerable identity (>74% nt identity to GLRaV-3. A nearly complete genome sequence (18,126 nts; GLRaV-3 isolate Kat255b; GenBank: MH521109) was obtained from a ‘Katelin’ vine of Canadian origin in the FPS pipeline. This sequence had 88% nt identity (99% query cover) to GLRaV-3 isolate Rod96 (GenBank: KY707825). Another GLRaV-3-like genome sequence (18,456 nts; GLRaV-3 isolate Gre233; GenBank: MH521105) was obtained from a ‘Grüner Veltliner’ (Green Veltliner) vine located at the NCGR. This sequence had 90% nt identity (96% query cover) with GLRaV-3 isolate Trc139 (GenBank: KY764332). An 18,398 nt sequence (GLRaV-3 isolate Mar239; GenBank: MH521114) was obtained from a domestic selection, ‘Marzemino’ variety, in the NCGR; this sequence had 90% nt identity (97% query cover) to isolate Trc139. Additionaly, isolate Mar239 had 99% pairwise identity to isolate Gre233; thus, representing the same GLRaV-3 variant. Finally, the sample ON936 generated a 18,599 nt sequence (GLRaV-3 isolate Vdl; GenBank: MK032068), confirming the result and sequencing data previously obtained during the Canadian study. GLRaV-3 isolate Vdl had only 75% nt identity (87% query cover) to GLRaV-3 isolate GH11 (GenBank: JQ655295). All four identified isolates did not cluster with other GLRaV-3 isolates with assigned group (Fig 4) based on the phylogenetic analysis.

To investigate the genetic diversity of GLRaV-3 isolates present in the NCGR, FPS pipeline and selected grape-growing regions of California, several grapevine samples were analyzed by HTS. Contigs of variable length (4,000 to 18,500 nts) but with high sequence identity (99% nt identity) to seven known GLRaV-3 isolates were identified in the NCGR (3138–07, GH24, GH11, Trc139, GH30, TRAJ-BR, and TC-BR) and the FPS pipeline (TRAJ-BR, WA-MR, ISAB-BR, GH24, LN, TC-BR, and 623); five different GLRaV-3 isolates (I-LR101, WA-MR, Rod96, TC-BR, and LN) were detected in samples collected in selected grape-growing regions of California (Table 4). Following current GLRaV-3 classification, isolates clustering with genetic variant groups I, II, III and VII were identified in the FPS pipeline; genetic variant groups I, II, VI, VII, and variants with no assigned group were identified in the NCGR collection; and groups I-III and IX were present in samples from commercial vineyards in California. Overall, 13 samples had mixed infection by two different GLRaV-3 variants.

**Table 4:** Diversity of grapevine leafroll-associated virus 3 (GLRaV-3) isolates in different grapevine populations.

Sample^a^	GLRaV-3 Isolate	GenBank Accession	GLRaV-3 Group^b^
FPS pipeline 1	TRAJ-BR	KX756669.1	I
FPS pipeline 1	ISAB-BR	KX701860.1	II
FPS pipeline 2	TRAJ-BR	KX756669.1	I
FPS pipeline 3	WA-MR	GU983863.1	I
FPS pipeline 4	TRAJ-BR	KX756669.1	I
FPS pipeline 4	TC-BR	KX756668.1	II
FPS pipeline 5	ISAB-BR	KX701860.1	II
FPS pipeline 6	GH24	KM058745.1	VII
FPS pipeline 7	LN	JQ423939.1	III
FPS pipeline 8	WA-MR	GU983863.1	I
FPS pipeline 9	TC-BR	KX756668.1	II
FPS pipeline 10	623	GQ352632.1	II
FPS pipeline 11	TC-BR	KX756668.1	II
FPS pipeline 11	LN	JQ423939.1	III
FPS pipeline 12	GH24	KM058745.1	VII
FPS pipeline 13	TRAJ-BR	KX756669.1	I
FPS pipeline 13	623	GQ352632.1	II
FPS pipeline 14	TC-BR	KX756668.1	II
FPS pipeline 14	TRAJ-BR	KX756669.1	I
NCGR 1	3138–07	JX559645.1	I
NCGR 2	GH24	KM058745.1	VII
NCGR 2	TRAJ-BR	KX756669.1	I
NCGR 3	GH11	JQ655295.1	VI
NCGR 4	GH11	JQ655295.1	VI
NCGR 5	Trc139	KY764332.1	NAG
NCGR 5	3138–07	JX559645.1	I
NCGR 6	Trc139	KY764332.1	NAG
NCGR 7	GH30	JQ655296.1	VI
NCGR 7	TRAJ-BR	KX756669.1	I
NCGR 8	Trc139	KY764332.1	NAG
NCGR 9	TRAJ-BR	KX756669.1	I
NCGR 10	Trc139	KY764332.1	NAG
NCGR 11	GH30	JQ655296.1	VI
NCGR 12	GH11	JQ655295.1	VI
NCGR 13	GH11	JQ655295.1	VI
NCGR 13	TC-BR	KX756668.1	II
NCGR 14	GH30	JQ655296.1	VI
NCGR 15	TC-BR	KX756668.1	II
San Luis Obispo, CA 1	GLRaV-3-I-LR101	KY886362.1	III
San Luis Obispo, CA 2	WA-MR	GU983863.1	I
San Luis Obispo, CA 2	TC-BR	KX756668.1	II
San Luis Obispo, CA 3	Rod96	KY707825.1	IX
San Luis Obispo, CA 4	Rod96	KY707825.1	IX
San Luis Obispo, CA 4	WA-MR	GU983863.1	I
San Luis Obispo, CA 5	TC-BR	KX756668.1	II
Sonoma, CA 6	Rod96	KY707825.1	IX
Sonoma, CA 7	GLRaV-3-I-LR101	MF186605.1	III
Sonoma, CA 7	TC-BR	KX756668.1	II
Sonoma, CA 8	LN	JQ423939.1	III
Monterey, CA 9	LN	JQ423939.1	III
Monterey, CA 10	Rod96	KY707825.1	IX
Napa, CA 11	Rod96	KY707825.1	IX
Napa, CA 11	LN	JQ423939.1	III
Central Sierra, CA 12	TC-BR	KX756668.1	II
Central Sierra, CA 13	LN	JQ423939.1	III

Identification of GLRaV-3 isolates was determined using the BLASTn program with sequences matching 99%.

^a^Foundation Plant Services (FPS); National Clonal Germplasm Repository (NCGR); California (CA).

^b^No assigned group (NAG).

### GLRaV-3 survey in New Zealand

For the 61 samples analyzed at The New Zealand Institute for Plant and Food Research Limited, DAS-ELISA and FPST results correlated (S4 Table), identifying 19 plants free of GLRaV-3 and 42 vines infected with the virus; positive samples included plants from the field trial for which the infection status was known. The FPST assay yielded C_T_ values ranging from 18.6 to 30.8 (mean of 23.1) from GLRaV-3-positive samples. The agreement between detection assays obtained in New Zealand is similar to the results generated at FPS.

## Discussion

In this study, we developed a new GLRaV-3 RT-qPCR assay that can detect diverse GLRaV-3 variants. The assay (called FPST) was validated using a two-step strategy, first using a small set of samples, followed by a large sample scale. Grapevine samples used for validation corresponded to domestic selections or foreign plants originating from 46 different countries in five continents. The GLRaV-3-status of these plants was also confirmed by a DAS-ELISA test and in a few cases by HTS. A broad and deep sampling design was used to maximize the chances for a representative set of GLRaV-3 variants. It was observed that the FPST assay not only detected all known GLRaV-3 variants, but also the newly identified genetically divergent variants (e.g. GH24 and NZ2). Additionally, an 18S rRNA assay [18] was used to guard against false negative results.

Our terminal assay (FPST) adds to the current library of available GLRaV-3 RT-qPCR assays [13,18,28]. Most of these already-published assays target the relatively conserved CP or HSP70h genes of the virus; in this FPST assay, primers and probes target the 3’ UTR. A small section in the 3’ terminal region of the GLRaV-3 genome was found conserved among variants known to date and was selected as the target for amplification. Previous studies on genetic diversity and diagnosis of GLRaV-3 have suggested that the 3’ end region of the virus is suitable for developing a detection assay [10,28]. The FPST assay developed uses six different primers, four forward and two reverse, and two MGB probes. During preliminary work, each probe was labeled with a different reporter (i.e. FAM and VIC) in order to identify GLRaV-3 variants and exclude any possibility of interference between probes. The number of primers and probes used in this assay may appear excessive for a single PCR reaction; however, they are essential to reduce the risk of false negatives because of nucleotide mismatch. The new FPST assay was tested against a genetically diverse set of distinct variants representing different variant groups, including single-variant infections during the small-scale screening. The FPST assay yielded C_T_ values ranging from 17.4 to 30 (mean of 21.2) from GLRaV-3-positive samples, with an high efficiency (110.91%), as calculated from the standard curve. In the few cases (18 samples) where high C_T_ values (35.5 to 39.5) were detected (S5 Table), samples were re-tested by the FPST assay and end-point RT-PCR (for details about this assay see [29]), and confirmed to be negative. We hypothesize that this amplification was caused by cross-contamination. Therefore, any amplification after 30 cycles should be further investigated and confirmed. Lastly, the FPST assay was evaluated using RNA extracts prepared from grapevines infected with GLRaV-1, GLRaV-2, GLRaV-4 and its strains (5, 6 and 9), which did not produce any amplification product indicating that this assay is specific for GLRaV-3.

All seven grapevines belonging to the Australian collection and displaying mild leaf roll symptoms tested positive by the FPST assay. These symptoms are associated with the infection by GLRaV-3m (GenBank: JX266782) [22], a divergent variant lacking the 3’ terminal region where the FPST probes and reverse primers bind. To investigate this unexpected result, RNA from the original source of GLRaV-3m (‘Sauvignon Blanc’ vine in the Adelaide Hills, South Australia) was analyzed. Using a specific forward primer for GLRaV-3m in combination with a reverse primer included in the FPST assay, the 3’ end of GLRaV-3m was extended (S1 Fig); thus, concluding that the sequence deposited in GenBank is partial, and the GLRaV-3m genome contains the region targeted by the FPST assay.

Our in-house developed DAS-ELISA assay was employed to test all plant samples used in the large-scale screening in the fall when the virus titer was the highest in the vine, and thus increasing the chance of accurate detection by the serological test. Results from the DAS-ELISA and the FPST assays were compared side-by-side to verify the accuracy and efficiency of the assay. The test results from the two assays were in 100% accordance. Agreement was obtained from the positive and negative samples, suggesting that both assays have the same specificity. The presence of GLD symptoms by GLRaV-3 variants could not be correlated with the virus because of the possibility of multiple infections by other GLRaV species. Additionally, as mentioned before, samples were collected late in the growing season with plants displaying certain degrees of senescence, thus, masking GLD symptoms.

We investigated the reproducibility of results generated by the FPST assay during the large-scale testing through a small survey in New Zealand. Subsequently, an agreement was obtained from the negative and positive grapevine samples for both the FPST assay and the DAS-ELISA. Based on these results, we predict that successful detection of GLRaV-3 by the FPST assay is fully replicable independently of the diagnostic lab conducting the analysis. Consequently, any lab around the world working with grapevine viruses can easily obtain the FPST primers and probes, which is not feasible with ELISA antibodies. An extra advantage of the FPST assay is the compatibility with pre-established analyses for other grapevine viruses. Grapevine red blotch virus (GRBV) [30] is an important virus pathogen of grapevine, because of the lack of serological assays, GRBV screening is performed by PCR-based assays; thus, detection of GLRaV-3 and GRBV can be carried out together using RT-qPCR, improving the high throughput sample processing efficiency.

RNA viruses have a high mutation rate and as a consequence extensive genetic diversity. In the case of GLRaV-3, the genetic diversity is not fully understood, mainly because the lack of sequence data. To address this information gap, we are searching for and characterizing genetically diverse variants of GLRaV-3 to expand sequence databases. To date, we have submitted near-complete genomes of 37 GLRaV-3 isolates to the GenBank. As a result of this study, three new distinct GLRaV-3 variants (represented by the isolates Kat255b, Gre233, Mar239 and Vdl) were identified using a hierarchical testing structure, which involved a combination of RT-qPCR assays along with HTS analysis. The phylogenetic analysis suggests that these novel GLRaV-3 variants do not belong to any previously known group, instead represent potentially new subclades with other highly divergent variants (Fig 4); thus, characterization of more GLRaV-3 isolates could provide an answer to this inquiry. On the other hand, it was observed that five GLRaV-3 isolates (NZ2, Cha141, Cha137, Cha138 and Trc139) clustered and formed an independent GLRaV-3 lineage in the phylogram; the pairwise identity between such isolates was 99%. Following the nomenclature proposed by Maree et al. [5] and given the low sequence identity of these five isolates with other GLRaV-3 variants (average 65%), we propose the creation of the group X (Fig 4).

The GLRaV-3 genetic diversity present in the NCGR, the FPS pipeline and grape-growing regions in California was investigated (Table 4), demonstrating that the NCGR samples included more diversity (at least five different groups) than the selected grape-growing regions of California and the FPS pipeline samples (four groups each). However, this higher diversity can be explained in the vast collection of genetically diverse germplasm present in the NCGR. Finally, mixed infections by different GLRaV-3 variants were identified, which might hinder the discovery of unknown variants.

The extensive validation presented here suggests that the new terminal assay (FPST) is sensitive and gives reproducible results. This new tool can be employed in routine diagnostic tests reducing the risk of GLRaV-3 spreading through infected grapevines as a result of false negative test results. The FPST assay detected all known variants to date, including isolates from different groups and supergroups of GLRaV-3, as well as the divergent variants pending group designation. The availability of additional GLRaV-3 genome sequences in the future will aid in further characterizing the genetic diversity, and in ascertaining whether any further diversity is likely to be detected by the FPST assay.

## Supporting information

S1 Table Grapevine samples included in the large-scale testing. (XLSX)

S2 Table GLRaV-3 sequences and GenBank accession numbers included in the phylogenetic analysis. (XLSX)

S3 Table Positive samples identified during the DAS-ELISA and FPST RT-qPCR testing. (XLSX)

S4 Table GLRaV-3 survey conducted at The New Zealand Institute for Plant and Food Research Limited. (XLSX)

S5 Table Samples that produced high CT values (>30) during the large-scale testing and were re-tested by the FPST assay and end-point RT-PCR. (XLSX)

S1 Fig Amplification and extension of the 3’ end of GLRaV-3m. (TIF)
