# Supplementary figures and images for: Characterization of grapevine leafroll-associated virus 3 genetic variants and application towards RT-qPCR assay design
Source: PLoS One. 2018 Dec 12;13(12):e0208862. doi: 10.1371/journal.pone.0208862 (PMC6291115; doi:10.1371/journal.pone.0208862)

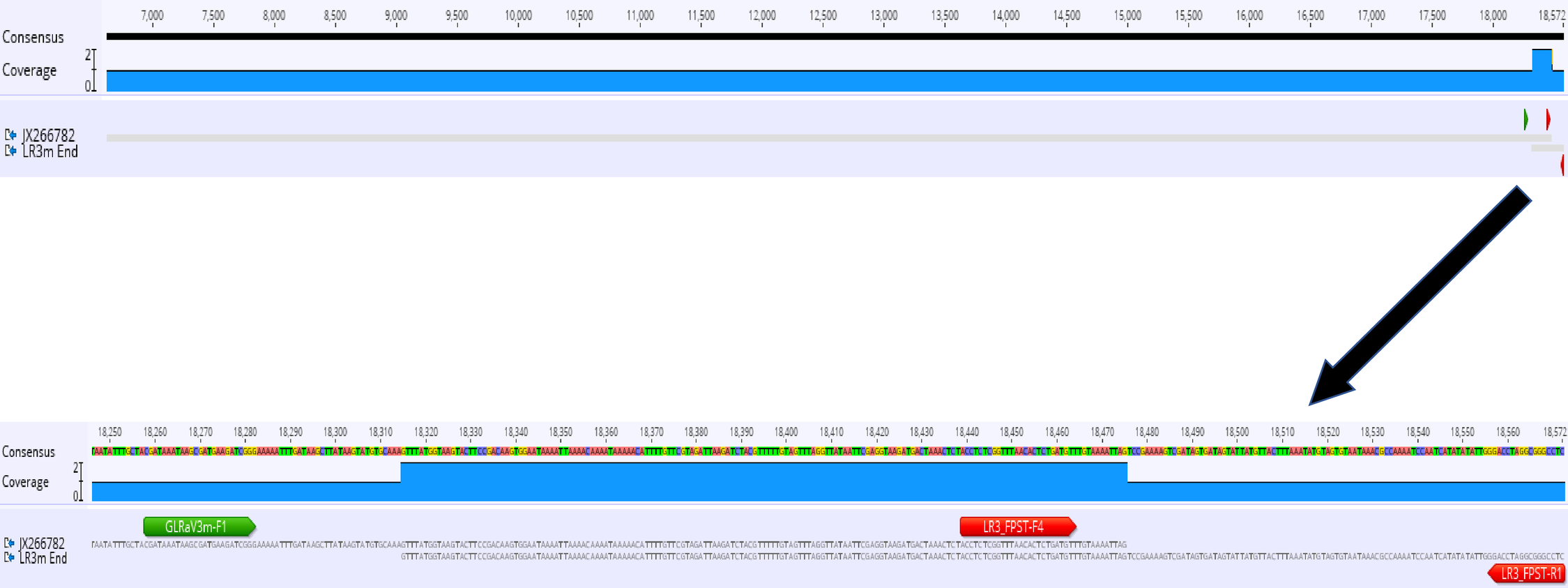

Supplement: S1 Fig — (TIF) [file pone.0208862.s006.tif]
